# Three cases of multi-generational Gaucher disease and colon cancer from an Ashkenazi Jewish family: A lesson for cascade screening

**DOI:** 10.1016/j.ymgmr.2019.01.001

**Published:** 2019-01-04

**Authors:** Fady Hannah-Shmouni, Dominick Amato

**Affiliations:** a Clinical Biochemical Genetics, Division of Clinical and Metabolic Genetics, The Hospital for Sick Children, University of Toronto, Canada; b Mount Sinai Hospital, University of Toronto, Toronto, ON, Canada; c Department of Medicine, Mount Sinai Hospital, 60 Murray Street, Room L-315, Box 34, Toronto, ON M5T 3L9, Canada

## Abstract

Gaucher disease (GD) is one of the commonest lysosomal storage diseases that is inherited in an autosomal recessive manner and affects 1 in 50,000 to 100,000 people in the general population. The frequency is much higher (1 in 500 to 1000) in people of Ashkenazi Jewish heritage due to a founder effect. GD is caused by decreased or absent activity of β-glucosidase with subsequent accumulation of the substrate glucosylceramide in macrophages due to genetic alterations in the *GBA* gene. These often accumulate in the spleen, liver and bone marrow. Three types exist, with type 1 being the most common, also referred to as non-neuronopathic GD. A broad clinical spectrum exists; patients of any age may manifest with hepatosplenomegaly, anaemia, thrombocytopenia, lung disease, bone abnormalities or may remain asymptomatic throughout their lifespan. Multi-generational disease does not usually occur because the risk of disease with each pregnancy, presuming both parents are carriers of the condition, is 25%. Herein, we report an Ashkenazi Jewish family with multi-generational GD type 1 and multigenerational colon cancer in the same three individuals, and reinforce the importance of cascade screening in families with genetic conditions.

## 1 Background

Gaucher disease (GD) is one of the commonest lysosomal storage diseases that is inherited in an autosomal recessive manner and affects 1 in 50,000 to 100,000 people in the general population. The frequency is much higher (1 in 500 to 1000) in people of Ashkenazi Jewish heritage due to a founder effect. Thus, we sought to report on this finding to reinforce the importance of cascade screening in families with genetic conditions.

## 2 Case presentation

**Fig. 1:**
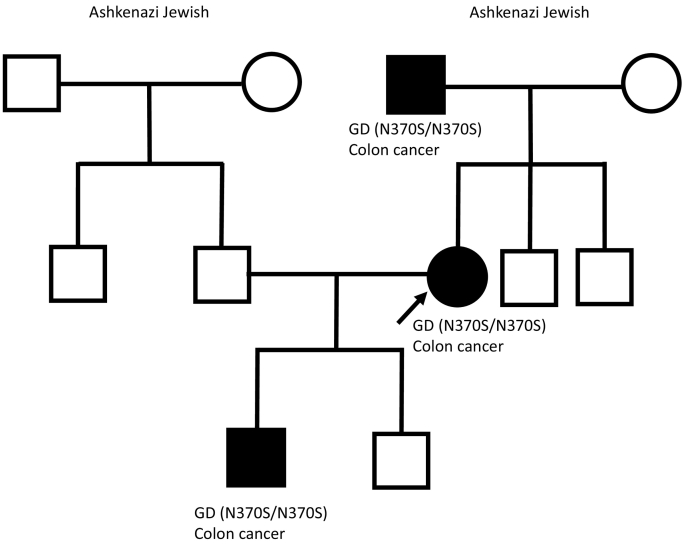
Pedigree demonstrating multi-generational Gaucher disease and colon cancer from an Ashkenazi Jewish family. GD: Gaucher disease.

A 66-year-old female from an Ashkenazi Jewish family with three successive generations of GD type 1 (Fig. 1) presented for evaluation of GD. Her past medical history was notable for osteopenia and gallstones. One of her two sons had both colon cancer and GD discovered in his 30s after evaluation of easy bruising, thrombocytopenia and splenomegaly. Her father had colon cancer, which was successfully resected; he was diagnosed with GD in his 30s after evaluation of thrombocytopenia and splenomegaly, requiring splenectomy. In her 50s, she had colon cancer that was resected with pathology showing Gaucher cells. She was otherwise asymptomatic with no significant medical or social history. She denied bone pains, bone fractures, easy bruising, bleeding, fatigue and weight loss. The family was tested for familial forms of colon cancer, such as Lynch syndrome, which was noncontributory. On examination, her pulse rate was regular but low at 42 beats per minute. There was no evidence of hepatosplenomegaly or purpura. There was evidence of mild cervical dystonia, with her neck ratcheting to the left with several movements.

Her complete blood count was within reference range. Testing for β-glucosidase activity showed reduced levels (2 nmol/h/mg, normal 8–16), confirming the suspicion for GD. A direct gene analysis for the common 9 pathogenic variants in the *GBA* gene was performed; a homozygous pathogenic variant in *GBA* (N370S/N370S) was detected, confirming GD. Biomarker analyses of GD that reflect excess lipid storage revealed elevated activity levels of angiotensin converting enzyme (ACE; 66, 9–63 U/L), chitotriosidase (CHITO; 1603, 4–120 nmoles/h/ml) and glucosylsphingosine (lyso-Gb1; 164, <10 ng/mL). Whole exome sequencing had been done previously on this family by the laboratory of Dr. Steven Gallinger (personal communication), and no disease-causing variants relating to colon cancer were found.

ECG revealed a left bundle branch block and bradycardia. MRI of the lumbar spine demonstrated marrow infiltration, with a bone marrow burden score of 6/16. MRI of the abdomen revealed a normal liver volume of 1300 mL and mild splenomegaly of 262 mL (normal volume for her weight would be ~150 mL). Confounding diagnoses that may present with similar clinical and biochemical features include hematologic malignancies such as lymphomas and leukaemias, which must be ruled out.

Successful treatments exist for GD type 1 and include enzyme replacement therapy (ERT) and glucosylceramide synthase inhibitors. Therapy is indicated for patients with type 1 GD who exhibit clinical signs and symptoms of the disease, including anaemia, thrombocytopenia, skeletal disease, or visceromegaly. Our patient did not meet criteria for therapy. Presymptomatic treatment remains controversial. To date, the patient remains asymptomatic with no clinical or radiographic evidence of worsening hepatosplenomegaly, anaemia, thrombocytopenia, lung disease, or major bone abnormalities.

## 3 Discussion

Gaucher disease (GD) is a multisystemic inherited metabolic disease that results from genetic alterations in the *GBA* gene leading to decreased β-glucosidase activity and subsequent accumulation of the substrate glucosylceramide in macrophages (known as Gaucher cells) [1]. This accumulation occurs in a variety of organs, including the spleen, liver and bone marrow leading to hepatosplenomegaly, anaemia, thrombocytopenia, lung disease, and bone abnormalities [1]. Herein, we describe a multi-generational Ashkenazi Jewish family with GD type 1.

GD is one of the commonest lysosomal storage diseases, inherited in an autosomal recessive manner and affects 1 in 50,000 to 100,000 people in the general population [2,3]. The frequency is much higher (1 in 500 to 1000) in people of Ashkenazi Jewish heritage [4,5]. The pathogenic variant N370S of *GBA* is particularly frequent in the Ashkenazi Jewish population (q approximately 0.03), while 84GG insertion (q approximately 0.003) occurs exclusively in this population [6]. Multi-generational disease does not usually occur because the risk of disease with each pregnancy, presuming both parents are carriers of the condition, is 25%. However, multi-generational disease should be suspected in founder populations, such as the Ashkenazi Jewish [4,5], and cascade screening of family members offered irrespective of age or symptoms.

As demonstrated in this case, the signs and symptoms of GD may go unnoticed for several decades or may remain unidentified across a patient's lifespan. Contrary to previous reports that patients of Ashkenazi Jewish descent with the common homozygosity of pathogenic variant N370S are asymptomatic throughout life and never come to medical attention, recent evidence suggests that the majority present with symptoms and disease progression in their lifespan [7]. Common manifestations of GD include easy bruising, bleeding, bone pains, splenomegaly, thrombocytopenia, anaemia, or chronic fatigue, with or without a positive family history [8]. Hyperferritinaemia and gammopathy are frequent biochemical abnormalities seen in GD [8].

The diagnosis of GD is established through demonstration of decreased enzymatic activity of β-glucosidase (also known as glucocerebrosidase) and confirmed by the identification of bi-allelic disease-causing variants in *GBA* (also referred to as *GBA1*) through whole-gene sequencing [9]. Historically, bone marrow aspiration was used for diagnosis; however, this procedure should be reserved when other haematological disorders must be ruled out [10]. After disease confirmation, measurement of disease biomarkers that correlate with the extent of glucocerebroside storage improves diagnosis, monitoring of disease progression and optimizing therapy [9]. Such biomarkers include chitotriosidase, glucosylsphingosine (Lyso-Gb1), and angiotensin converting enzyme.

GD is associated with several systemic manifestations that influence quality of life. Bone abnormalities include osteopenia, osteoporosis, bone fractures and bone infarction, which may manifest clinically as bone crises or osteonecrosis. Central nervous system manifestations are associated with types 2 and 3 GD, although recent evidence suggests that carriers of a disease-causing variant in *GBA* or patients with bi-allelic disease-causing variants leading to type 1 are at an increased risk of parkinsonism [11,12]. A recent study showed that one in three Ashkenazi Jewish patients diagnosed with dementia with Lewy bodies were carriers of a pathogenic variant in *GBA* [13]. The liver is one of the major storage sites involved in GD pathogenesis, and various liver-related complications have been reported including hepatocellular carcinoma [14]. Other complications include growth failure in children, interstitial lung disease, pulmonary hypertension and arrhythmias. Colon cancer is not a known comorbidity of GD; interestingly, all three members with GD in our report had early-stage colon cancer that was cured following surgical resection. One may speculate a connection between GD and colon cancer in these three persons, who are the only ones in the pedigree with GD and the only ones with colon cancer. As discussed above, whole exome sequencing had been done previously on this family, and no disease-causing variants relating to colon cancer were found. Further studies addressing the genetic underpinnings of colon cancer in individuals with and without GD through a whole-genome or GWAS approach might reveal new genetic backgrounds for this rather common malignancy.

Several treatment options for patients with GD exist. Enzyme replacement therapy (ERT), which has been available since 1991 [15], is a well-established treatment modality with known efficacy and minimal toxicity [9]. ERT results in significant improvements in spleen volume, platelet count, hemoglobin level, and liver volume [16]. Several ERT options exist in the market. Oral substrate reduction therapy (e.g., miglustat, eliglustat) through the inhibition of glucosylceramide synthesis is becoming popular in the management of some patients with GD [9]. Common indications for therapy initiation include severe anaemia, severe thrombocytopenia, episodes of splenic infarcts, acute bone crises, radiographic evidence of joint destruction, chronic bone pain, significant hepatosplenomegaly, progressive pulmonary disease and growth restriction in children. Combined miglustat and ERT can be tried [17]. Nowadays, symptomatic management rarely involves splenectomy or allogeneic bone marrow transplantation [9]. Orthopaedic surgery, including arthroplasties, could be required for management of osteonecrosis [9]. Some patients may never require therapy; our patient's father, who had GD, died at age 96 without ever having received treatment.

Multigenerational GD has been reported in two separate Lebanese families [18,19], but in both families, there was a high degree of consanguinity. Kolodny et al. [20] reported an Ashkenazi Jewish family with three generations of GD; there was no mention of consanguinity, but there was compound heterozygosity in three of the individuals. There was also no mention of malignancy in any of the patients. In our family, to the best of their knowledge there is no consanguinity.

In conclusion, we present a unique case report of multi-generational GD in a family of Ashkenazi Jewish descent. Multi-generational disease should be suspected in founder populations, such as the Ashkenazi Jewish and cascade screening offered to all family members to avoid missing a diagnosis.

## Consent

Written informed consent was obtained from the patient for the publication of this case report and any potentially-identifying information/images.

## References

[1] Pastores G.M., Hughes D.A., Disease Gaucher, Adam M.P., Ardinger H.H., Pagon R.A., Wallace S.E., Bean L.J.H., Stephens K. (1993). GeneReviews((R)). Seattle (WA).

[2] Meikle P.J., Hopwood J.J., Clague A.E., Carey W.F. (1999). Prevalence of lysosomal storage disorders. JAMA.

[3] Poorthuis B.J., Wevers R.A., Kleijer W.J., Groener J.E., de Jong J.G., van Weely S. (1999). The frequency of lysosomal storage diseases in the Netherlands. Hum. Genet..

[4] Zimran A., Gelbart T., Westwood B., Grabowski G.A., Beutler E. (1991). High frequency of the Gaucher disease mutation at nucleotide 1226 among Ashkenazi Jews. Am. J. Hum. Genet..

[5] Beutler E., Nguyen N.J., Henneberger M.W., Smolec J.M., McPherson R.A., West C. (1993). Gaucher disease: gene frequencies in the Ashkenazi Jewish population. Am. J. Hum. Genet..

[6] Diaz G.A., Gelb B.D., Risch N., Nygaard T.G., Frisch A., Cohen I.J. (2000). Gaucher disease: the origins of the Ashkenazi Jewish N370S and 84GG acid beta-glucosidase mutations. Am. J. Hum. Genet..

[7] Balwani M., Fuerstman L., Kornreich R., Edelmann L., Desnick R.J. (2010). Type 1 Gaucher disease: significant disease manifestations in "asymptomatic" homozygotes. Arch. Intern. Med..

[8] Mehta A., Belmatoug N., Bembi B., Deegan P., Elstein D., Goker-Alpan O. (2017). Exploring the patient journey to diagnosis of Gaucher disease from the perspective of 212 patients with Gaucher disease and 16 Gaucher expert physicians. Mol. Genet. Metab..

[9] Revel-Vilk S., Szer J., Mehta A., Zimran A. (2018). How we manage Gaucher Disease in the era of choices. Br. J. Haematol..

[10] Beutler E., Saven A. (1990). Misuse of marrow examination in the diagnosis of Gaucher disease. Blood.

[11] Aflaki E., Westbroek W., Sidransky E. (2017). The Complicated relationship between Gaucher disease and Parkinsonism: insights from a rare disease. Neuron.

[12] Neudorfer O., Giladi N., Elstein D., Abrahamov A., Turezkite T., Aghai E. (1996). Occurrence of Parkinson's syndrome in type I Gaucher disease. QJM.

[13] Shiner T., Mirelman A., Gana Weisz M., Bar-Shira A., Ash E., Cialic R. (2016). High frequency of GBA gene mutations in dementia with lewy bodies among Ashkenazi Jews. JAMA Neurol..

[14] Regenboog M., van Dussen L., Verheij J., Weinreb N.J., Santosa D., Vom Dahl S. (2018). Hepatocellular carcinoma in Gaucher disease: an international case series. J. Inherit. Metab. Dis..

[15] Barton N.W., Brady R.O., Dambrosia J.M., Di Bisceglie A.M., Doppelt S.H., Hill S.C. (1991). Replacement therapy for inherited enzyme deficiency—macrophage-targeted glucocerebrosidase for Gaucher's disease. N. Engl. J. Med..

[16] Mistry P.K., Lukina E., Ben Turkia H., Amato D., Baris H., Dasouki M. (2015). Effect of oral eliglustat on splenomegaly in patients with Gaucher disease type 1: the ENGAGE randomized clinical trial. JAMA.

[17] Amato D., Patterson M.A. (2018). Combined miglustat and enzyme replacement therapy in two patients with type 1 Gaucher disease: two case reports. J. Med. Case Rep..

[18] Shamseddine A., Taher A., Fakhani S., Zhang M., Scott C.R., Habbal M.Z. (2004). Novel mutation, L371V, causing multigenerational Gaucher disease in a Lebanese family. Am. J. Med. Genet..

[19] El-Zahabi L.M., Makarem J., Habbal Z., Otrock Z.K., Taher A., Shamseddine A. (2007). Gaucher disease: different clinical manifestations associated with a rare mutation (R48W) in a Lebanese family. Molec Genet Metab..

[20] Kolodny E.H., Firon N., Eyal N., Horowitz M. (1990). Mutation analysis of an Ashkenazi Jewish family with Gaucher disease in three successive generations. Am. J. Med. Genet..

